# Metabolic Syndrome among Emirati Adolescents: A School-Based Study

**DOI:** 10.1371/journal.pone.0056159

**Published:** 2013-02-13

**Authors:** Aaesha E. Mehairi, Aysha A. Khouri, Muna M. Naqbi, Shamma J. Muhairi, Fatima A. Maskari, Nico Nagelkerke, Syed M. Shah

**Affiliations:** 1 Department of Family Medicine, Faculty of Medicine and Health Sciences, United Arab Emirates University, Al Ain, United Arab Emirates; 2 College of Medicine and Health Sciences, Institute of Public Health, United Arab Emirates University, Al Ain, United Arab Emirates; CUNY, United States of America

## Abstract

**Objectives:**

Population-based data on metabolic syndrome (MetS) among children is lacking in the United Arab Emirates which has among the highest rates of diabetes in the world. In this study we determined the prevalence of MetS and its correlates in a sample of adolescents.

**Materials and Methods:**

A cross-sectional school-based study was conducted on 1,018 adolescents (48.4% girls) aged 12–18 years from Al Ain Abu Dhabi Emirates. A self-administered questionnaire was used to assess socio-demographic characteristics, physical activity and dietary habits. Blood pressure, height, weight, waist circumference, fasting glucose, HDL-cholesterol and triglycerides were measured. MetS was defined using the International Diabetes Federation (IDF) criteria.

**Results:**

The prevalence of metabolic syndrome was 13%. Boys compared to girls were more likely to have MetS (21% vs. 4%, odds ratio [OR]: 6.57, 95%CI: 4.01 to 10.75). The prevalence of MetS increased with increase in body mass index and reached 59 percent in obese boys. After multivariable adjustment boys who were overweight (adjusted OR: 2.72 [1.37 to 5.35]), or obese (AOR: 12.70 [7.31 to 22.05]), or spent two or more than two hours on screen in a day (AOR: 1.65 [1.01 to 2.69) were more likely to have MetS. Girls who were overweight (AOR: 4.23 [1.32 to 13.62]) or obese (AOR: 8.32 [2.73 to 25.32]) were more likely to have MetS.

**Conclusions:**

The prevalence of MetS is high among UAE boys. Population-based strategies are needed to address the high burden of metabolic syndrome targeted at the identified risk factors.

## Introduction

Metabolic syndrome (MetS) is defined as a pattern of metabolic disturbances including central obesity, hyperglycemia, dyslipidemia, and hypertension. Although it has been questioned whether MetS constitutes a true “syndrome”, it does appear to be a useful summary measure of atherosclerotic cardiovascular disease in adults [1]. Studies also show that pediatric metabolic syndrome is a significant predictor of metabolic syndrome, type 2diabetes and cardiovascular disease in adults [2.3].

Obesity is a common cause of insulin resistance in children [4]. As both obesity and insulin resistance are constituent components of MetS, MetS is rapidly increasing in prevalence worldwide in association with rising childhood obesity [5]. In the United Arab Emirates (UAE) rapid socioeconomic growth has resulted in profound lifestyle changes including sedentary behaviors, Westernized diets and increased energy intake. A recent nationwide study [6] on the prevalence of overweight and obesity, dietary and activity patterns in UAE population showed that the average daily total caloric intake was the highest for adolescent Emirati boys. Almost one third (34%) of UAE youth between ages of 12 and 18 years were already overweight or obese.

We conducted this study in Al Ain, Abu Dhabi Emirates. Abu Dhabi is the largest of the seven emirates accounting 87% of the area that make up the United Arab Emirates. Al Ain (“the spring” in Arabic) is the second largest city, located approximately 160 kilometers east of the Abu Dhabi capital. Local Emiratis make up 33 per cent of the population in Al Ain. Despite the evidence that UAE has among the highest rates of diabetes in the world [7], population-based data on MetS prevalence in Emirati youth population is lacking. A comprehensive understanding of MetS in adolescent population may be important for the specific direction of prevention strategies because both the magnitude and the prevalence of childhood obesity have increased. Therefore, the aim of this research was to estimate the prevalence of metabolic syndrome and its correlates among Adolescents in Al Ain Abu Dhabi Emirates.

## Materials and Methods

**Figure 1:**
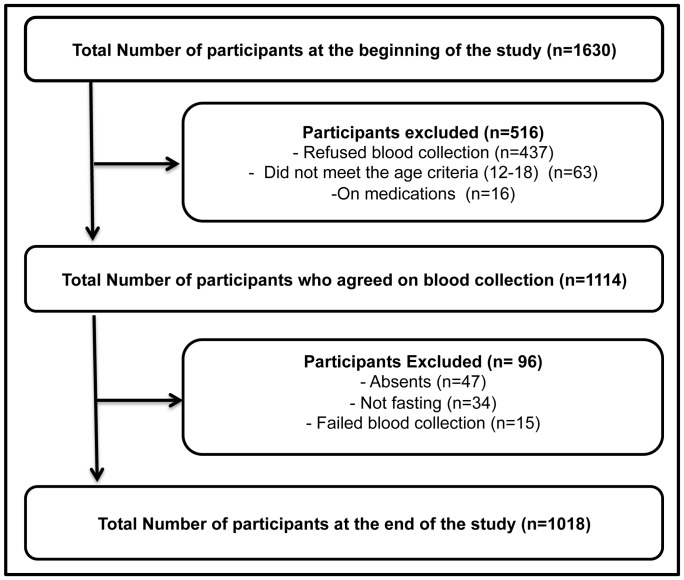
Enrolment process of study participants, youth aged 12 to 18 years (n-1018), Al Ain, United Arab Emirates. WC waist circumference, IFG impaired fasting glucose, HDL high density, Lipoprotein cholesterol, TG triglycerides, hypertension BP≥130/85.

The cross-sectional study was conducted in March and April 2010 in Al Ain Abu Dhabi, the largest and wealthiest of the 7 emirates of the United Arab Emirates (UAE). The sampling frame of primary sampling units (schools) were all schools enrolling adolescents aged 12 to 18 years in Al Ain. Out of 114 schools, 8 were randomly selected by using SPSS Software (version 19) program. Students within schools were sampled proportional to the enrollment size of each school to get a self-weighting sample. Out of 1630 students thus selected, 516 were excluded because they did not meet the inclusion criteria of the study i.e. older than 18 or less than 12 years (n = 63), had a disease and on medication (n = 16) or refused to give blood sample (n = 437). Out of 16 youth on medication 2 were using oral hypoglycemic drugs and 1 was using insulin to control the blood sugar level. Out of the participants (n = 1,114) who agreed to participate in the survey, 47 were absent from school at the time of survey, 34 were not fasting, 13 failed the blood collection process and 2 fainted during blood collection. The enrolment process has been shown in Figure 1. Thus the final sample for this study consisted of 1018 participants.

Our institutional ethics committee the Al Ain Medical District Human Research Ethics Committee approved this study. Informed written consent was taken from participating children. We also obtained written informed consents from parents on the behalf of the minors/children involved in our study.

Personal identification numbers were assigned to participant to maintain anonymity. The identification number confirmed consent status and linked students to their respective schools, clinical measurements and blood samples. Confidentiality of all subjects was fully protected and only aggregate data was reported and released.

The questionnaire was a pilot tested, self-administered one. Investigators were available if respondents needed clarification of particular question. The questionnaire was designed to be completed within a single 45-minute class period. We used already validated question in UAE population [6] and we used more objective questions whenever possible. The questions focused on obtaining demographic data, evaluating the risk factors for metabolic syndrome such as age, sex, socioeconomic status, ethnic group, life style (physical activity, sedentary behavior, and smoking), obesity and family history of diabetes, hypertension and dyslipidemia. Non-vegetarian food is not a part of daily consumption of food in UAE. We included few questions on eating habits including questions on fast food consumption, sugar-sweetened drinks and soda consumptions. The variables studied regarding fast food were related to 7-day recall of intake of commonly available meals such as beef burger, chicken burger, and chicken nugget with French fries consumed at one of the fast food restaurant outlets, such as McDonalds, Burger King etc. Local foods in the form of shawarma were also available at some fast food outlets containing more fat, free sugars and high sodium.. We did not include questions related to alcohol consumption. The short version of the International Physical Activity Questionnaire (IPAQ) was used to assess the physical activity status in the study participants [8].

A training workshop for all the selected school’s nurses was conducted by qualified trainers before data collection. This workshop covered standardized methods of taking anthropometric measurements and measuring blood pressures. Pictorial and written instructions were available for reference. Height was measured to the nearest 0.1 centimeters (cm) in an upright position without shoes by using a stadiometer (SECA). Waist circumference was also measured in centimeters (cm) by using an un-stretched measuring tape to measure the midpoint between the bottom of the rib cage and above the tip of the iliac crest. Weight was measured to the nearest 0.1 in kilogram (Kg) in a standing position without shoes and in light clothing by using a digital scale (SECA). Three readings of height, weight and waist circumference were taken and the average was used in analyses. Blood pressure (BP) was measured on the right arm of subjects seated and at rest for at least 5 minutes. Three consecutive measures were obtained at 1-minute intervals with a standard mercury sphygmomanometer with an appropriate cuff size, and the average of the last two readings was used in the analyses.

A five milliliter of venous blood sample was obtained from the subjects by qualified nurses using standardized tubes: Serum Separator Tube (SST) for lipid profile and fluorinated tube for fasting blood glucose. Before withdrawing blood, all participants were ensured to be fasting for at least eight hours. The samples were then sent to Tawam hospital laboratory where they underwent standardized quality controlled analysis. The fasting plasma glucose (FBS), high density lipoprotein-cholesterol (HDL-C) and triglycerides (TG) were analyzed by the DXC 800 Analyzer (Beckman Coulter, Fullerton, CA, USA) using the appropriate conventional laboratory reagents, enzymatic and calorimetric techniques.

We used waist circumference cut points established by Katzmarzyk *et al* [9] to identify abdominal obesity. The diagnostic criteria of MetS in children and adolescents, including waist circumference ≥90^th^ percentile or ≥94^th^ percentile (for youth aged 16 years or older), triglycerides levels ≥150 mg/dL(1.7 mmol/L), HDL-C <40 mg/dL(1.03 mmol/L) or <50 mg/dL (1.29 mmol/L) for female adolescents aged ≥16 years and older, fasting plasma glucose (FPG) levels of >100 mg/dL (5.6 mmol/L) and blood pressure (BP) ≥130/80 mmHg according to the International Diabetes Federation guidelines [10].

Body mass index (BMI) was calculated from weight (kg) divided by the squared height (m^2^). We used the international BMI age- and gender-specific cut-off points as shown in Table S1) to define overweight and obesity (based on pooled international data and linked to the widely used adult cut-off points of a BMI of 25 and 30 kg/m^2^) [11].

Screen time included computer game use, video and television. Subjects reported the number of hours per typical day in the past 30 days that they watched television (including videos) and or used a computer (including video games) during their free time. Screen hours were used to create a dichotomous variable of less than 2 hours or ≥2 hours.

Completed questionnaires, were checked for errors. Data was entered using Microsoft Access to reduce entry errors. Then it was analyzed using frequency and cross tabulations by using SPSS (version 19) and Stata (Stata Corp, College Station, TX) program version 11.1 for regression models with random effects. To explore the effects of non-participation, we compared age, gender, ethnicity and body mass index of participants (n = 1018) and non-participants (N = 437 who refused blood draw). Descriptive statistics were used for analysis. Continuous variables were summarized into means and standard deviations. Categorical variables were expressed in proportions and their 95% confidence intervals. Chi-square analysis was performed to test the association between categorical variables. The student t-test was used to compare means among groups. We also conducted a trend test of association across categories of body mass index and MetS (presented as p_trend_). P-values of <0.05 were considered significant. Univariate analysis was carried out to select variables for inclusion in a stepwise logistic regression (backward selection) of MetS on potential determinants. We conducted multivariate analysis using variables found significant (p<0.05) in univariate analysis and included age, screen time, BMI and LDL-C as continuous variables. We also addressed possible within-school clustering of MetS (using school as a random variable in the final multivariate model).

## Results

Of the 1018 participants, 48.4% were girls. The majority were UAE nationals (52%). General clinical characteristics of the study population are shown in table 1. There were no statistically significant differences in age, sex, ethnicity and body mass index between participants and those who refused blood draw. A high proportion of youth were overweight and obese (34.6%). More boys (39%) than girls (30%) were overweight or obese. A high proportion of boys (22%) had abdominal obesity compared to girls (4%). Overall 16% reported eating at a fast-food restaurant on more than three days during the last 7 days. More boys (18%) than girls (13%) consumed fast food. Almost half (49.1%) of study participants consumed soft drinks once or more in a day. More boys (60.7%) than girls (36.7%) consumed soft drinks. Only 5% of the participants reported zero screen time. More than half of the study participants (53.8%) spent ≥2 hours on screen. We also asked about the family history of diabetes. Out of 1018 study participants 141 did not know about the family history of diabetes.

**Table 1:** Characteristics of the study participants aged 12 to 18 years (n = 1018).

	All	With MetS	Without MetS	p value
N		140	878	
Age (Years)	15.4±1.8	15.2±1.8	15.4±1.7	0.155
Height (cm)	163.1±10.2	167.4±12.4	162.5±9.6	0.000
Weight (kg)	61.2±17.9	80.7±24.2	53.8±14.6	0.000
BMI (kg/m^2^)	22.9±6.1	29.0±10.1	21.9±4.6	0.000
Overweight/obese	34.60%	74.60%	30.60%	0.000
Waist circumference (cm)	72.3±13.1	89.0±17.9	69.7±10.1	0.000
Systolic BP (mmHg)	107.5±12.1	113.2±13.8	106.8±32.2	0.047
Diastolic BP (mmHg)	63.4±9.2	64.2±10.5	63.8±32.8	0.997
Fasting blood glucose (mmol/L)	5.0±0.5	5.3±0.5	4.9±0.5	0.000
Total cholesterol(mmol/L)	4.1±0.7	4.0±0.8	4.1±0.7	0.679
LDL cholesterol (mmol/L)	2.7±0.6	2.7±0.8	2.6±0.6	0.589
HDL cholesterol (mmol/L)	1.0±0.3	0.8±0.1	1.1±0.3	0.000
Triglycerides (mmol/L)	0.7±0.4	1.1±0.7	0.6±0.3	0.000

Data presented as means ± standrad deviation,

p-values are for tests comparing those with MetS and Without MetS.

BMI, body mass index.

There was no significant difference in prevalence of MetS among those who reported having a family history compared to those who did not report a family history as shown in Table 2. Waist circumference was significantly larger (p<0.001) in those with MetS compared to those without MetS. No statistically significant differences were noted in mean age, diastolic blood pressure, total and HDL-cholesterol between the two groups (Table 1). A high proportion of adolescents with MetS were overweight and obese (74.6%).

**Table 2:** Participants characteristics by Metabolic Syndrome (MetS), aged 12 to 18 years (n = 1018), Al Ain, UAE, 2010: univariate analysis.

	Boys				Girls			
Characteristics	All	With MetS			All	With MetS		
	n	n (%)	OR (95%CI)	p value	n	n (%)	OR (95%CI)	p value
Study population	522	114 (21.80)	6.57 (4.01–10.75)	0.000	49	20 (4.1)	1 (Reference)	
Age (Years)								
12 to 14 (reference)	158	33 (20.9)	1		153	9 (5.9)	1	
15 to 16	181	43 (23.8)	1.18 (070–1.97)	0.527	162	11 (6.8)	2.58 (1.04–6.36)	0.039
17 to 18	183	38 (20.8)	0.99 (0.59–1.68)	0.978	175	_	_	_
Age (continuous variable)			0.99 (0.88–1.11)	0.885			0.66 (0.51–0.84)	0.001
Family history of diabetes								
No	177	54 (20.7)	1		166	13 (4.9)	1	
yes	261	38 (21.5)	0.94 (0.59–1.62)	0.844	267	5 (3.0)	1.65 (0.57–4.71)	0.351
Father education								
No formal education (reference)	38	4 (10.5)	1		17	2 (11.8)		
Up to secondary level	203	49 (24.1)	2.70 (0.91–8.00)	0.072	184	6 (3.3)	0.25 (0.05–1.36)_	0.110
College/University	161	33 (20.5)	2.19 (0.72–6.61)	0.164	200	8 (4.0)	0.31 (0.06–1.60)	0.164
Mother education								
No formal education	61	14 (22.9)	1		38	2 (5.3)	1	
Up to secondary level	224	52 (23.2)	1.01 (0.52–1.98)	0.966	264	9 (3.4)	0.63 (0.13–3.05)	0.572
College/University	120	21 (17.5)	0.71 (0.33–1.52)	0.381	133	6 (4.5)	0.85 (0.16–4.39)	0.847
Physical Activity Score								
Low (reference)	126	27 (21.4)	1		224	9 (4.0)	1	
Moderate	143	27 (18.9)	0.85 (0.47–1.55)	0.603	155	7 (4.5)	1.13 (0.41–3.10)	0.813
High	246	59 (23.9)	1.16 (0.69–1.94)	0.580	104	4 (3.8)	0.95 (0.29–3.18)_	0.941
Cigarette smoking								
yes	165	39 (23.6)	1.16 (0.74–1.79)	0.520	18	_	_	_
No	355	75 (21.1)	1		472	20 (4.2)		
Hours on screen in past 7 days								
<2 Hours per day (reference)	294	53 (18.0)	1		165	11 (6.67)	1	
≥2 Hours	215	58 (26.9)	1.68 (1.10–2.56)	0.016	319	9 (2.8)	0.40 (0.16–1.01)	0.050
Total hours on screen			1.08 (1.00–1.15)	0.038			0.95 (0.80–1.83)	0.603
Fast Food in past 7 days								
No	118	25 (21.2)	1		136	5 (3.7)	1	
≥1 day	404	89 (22.0)	1.05 (0.64–1.73)	0.845	354	15 (4.2)	1.16 (0.41–3.25)	0.779
Carbonated soft drink consumption								
Less than once a day (reference)	205	42 (20.5)	1		310	10 (3.2)	1	
Once or more than once a day	317	72 (22.7)	1.14 (0.74–1.75)	0.548	180	10 (5.6)	1.76 (0.71–4.32)	0.214
Nationality								
Other Countries (reference)	258	50 (19.4)	1		225	8 (3.5)	1	
Emirati	262	64 (24.4)	1.34 (0.88–2.04)	0.165	265	12 (4.5)	1.28 (0.52–3.20)	0.588
LDL -cholesterol								
≥160 mg/Dl (8.9 mmol/L)	387	46 (27.2)	1.57 (1.02–2.41)	0.040	213	7 (3.3)	0.69 (0.27–1.76)	0.438
<160 mg/Dl	631	68 (19.3)	1		217	13 (4.7)	1	
Total LDL -Cholesterol			1.43 (1.05–1.95)	0.024			0.81 (0.37–1.75)	0.591
Nutritional Status (BMI)								
Not overweight/obese	312	27 (8.6)	1		333	6 (1.8)	1	
Overweight	84	17 (20.2)	2.68 (1.38–5.19)	0.004	91	6 (6.6)	3.85 (1.21–12.23)	0.022
Obese	125	69 (55.2)	13.0 (7.66–22.08)	0.000	66	8 (12.1)	7.51 (2.51–22.46)	0.000
			Ptrend = 0.000				Ptrend = 0.000	
BMI (continuous)			1.25 (1.19–1.31)	0.000			1.14 (1.06–1.23)	0.000

**Figure 2:**
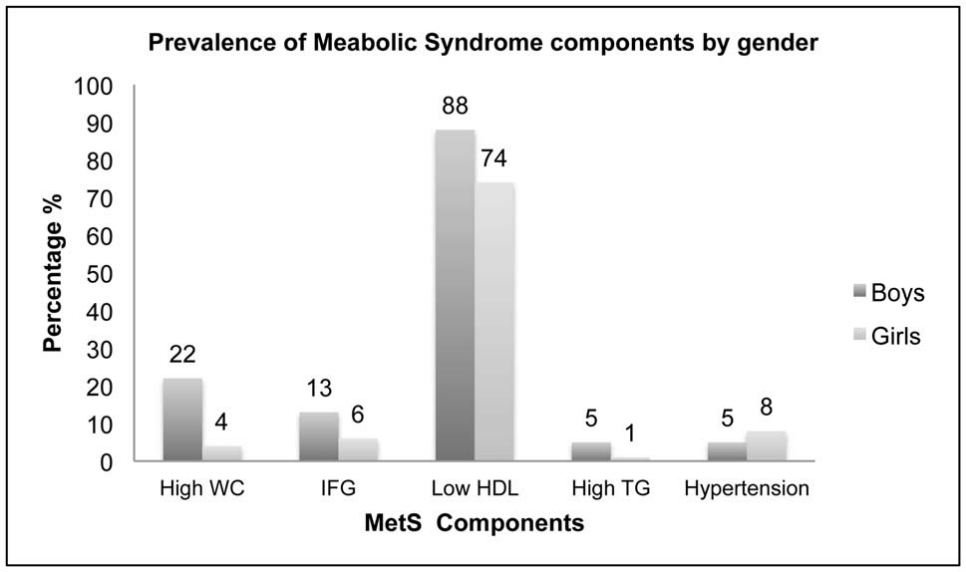
Prevalence of metabolic syndrome components by gender among youth aged 12 to 18 years (n = 1018), Al Ain, United Arab Emirates.

The overall prevalence of MetS was 13% according to the IDF criteria. MetS was more prevalent among boys (22%) than in girls (4%). Figure 2 shows the distribution of MetS and its components by gender.

**Figure 3:**
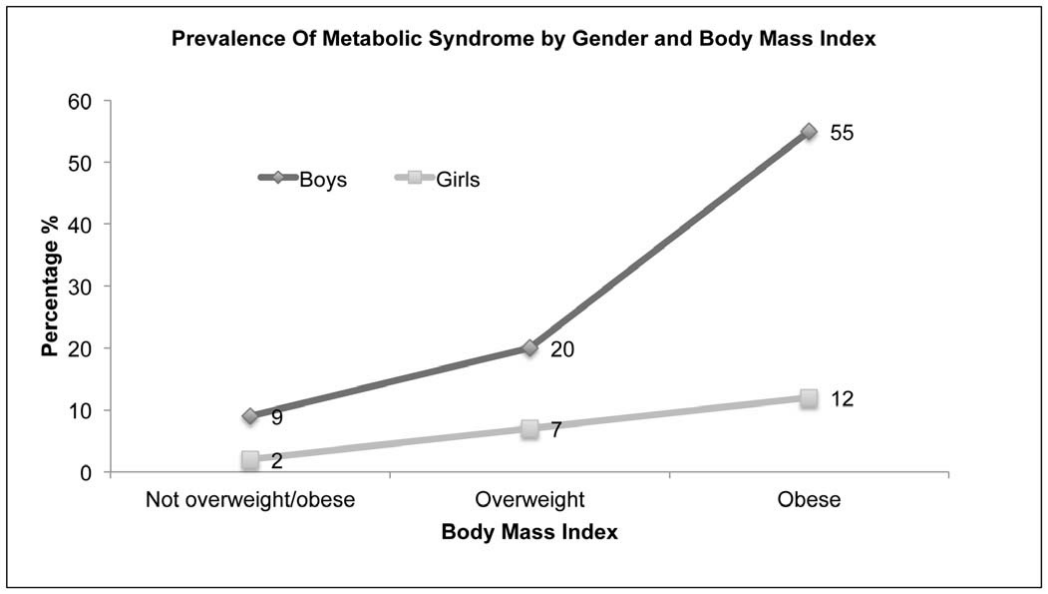
Prevalence of metabolic syndrome by gender and BMI among youth aged 12 to 18 years (n = 1018), Al Ain, United Arab Emirates.

Figure 3 shows the prevalence of MetS by BMI categories and gender. Prevalence of Mets increased with body mass index. There was a significant (p<0.05) linear relationship between MetS prevalence and BMI categories among boys and girls and its prevalence was extremely high (59%) among obese boys.

Table 2 shows the univariate analysis for correlates of MetS. Boys were more likely to have MetS than girls (OR: 6.57 [95% CI: 4.01 to 10.75]). Boys who reported more than 2 hours per day of screen time were found to have a high prevalence of metabolic syndrome (26.94%). In this study Emirati boys had a higher prevalence of MetS (24.4%) than their counterparts from other countries (19.4%).

Table 3 shows the results of multivariable analysis. Multivariable results show that obesity, screen time, and age were associated with MetS.

**Table 3:** Multivariate analysis: adjusted odds ratios of risk factors for Metabolic Syndrome (MetS) in youth aged 12 to 18 years (n = 1018), Al Ain, UAE.

Characteristic	Boys		Girls	
	With MetS		With MetS	
	Adjusted OR (95%CI)	p value	Adjusted OR (95%CI)	p value
Hours on screen (continuous variable)	1.08 (1.00–1.17)	0.050	0.99 (0.82–1.20)	0.974
Body mass index (kg/m2)	1.26 (1.20–1.33)	0.000	1.22 (1.12–1.33)	0.000
LDL-cholesterol (mmol/dl)	0.96 (0.67–1.36)	0.831	0.61 (0.25–1.49)	0.282

This model was adjusted for the age of child and school.

## Discussion

Our findings suggest that the metabolic syndrome is common among UAE adolescents and that, not surprisingly, its prevalence increases directly with the degree of obesity. Lower prevalence have been reported from neighboring countries, *viz*. 9.4% among children and youth aged 10 to 18 years in Saudi Arabia [12], 10.1% among Iranian youth aged 10 to 19 years [13], 7.4% among Egyptian adolescents aged 10 to 18 years [14] as well as the USA, where prevalence of 12% in Mexican-Americans, 10.4% in non-Hispanic whites, 13.4% in non-Hispanic blacks among youth aged 12 to 19 years [15] have been reported. However these studies used the NCEP ATP III definition of MetS. Using IDF criteria the prevalence was only 3.6% among Jordanian youth aged 16 to 18 years [16], 4.6% among Vietnamese youth aged 12 to 15 years [17] and 4.5% among US adolescents aged 12 to 17 years [18]. No population-based data have yet been reported from the UAE but a clinic-based study in 2005, recorded 44% prevalence of MetS in obese youth [19]. Clearly, the prevalence depends on what defining criteria are used for the MetS. There has been no international consensus reached as yet although attempts are ongoing [20].

Similar to others [21]–[22], we found a significant difference in MetS prevalence between boys (21.8%) and girls (4.1%). This may be related to hormonal differences, such as testosterone and sex- hormone binding globulin (SHBG) between genders [23]. More pragmatically, the higher energy and fat intake among males may well have contributed substantially to this difference.

We found strong correlations between overweight and obesity and the prevalence of MetS in both genders, and this has been supported by other studies [24], [25]. While this would seem logical, obesity being a constituent of MetS, and a recent study on obesity in adolescents reported that this group had a significant degree of insulin resistance, high TG and low HDL levels, which might strengthen this association [26].

Boys had a substantially higher prevalence of overweight and obesity than girls. The high prevalence of MetS in obese boys (59%) is a cause for concern. Childhood obesity track into adulthood and metabolic syndrome in childhood predicts adult cardiovascular disease [27]. Studies indicated that childhood obesity and glucose intolerance greatly increased the risk of premature death among Pima Indians [28].

Our data showed a significant independent association between screen time and MetS among boys. Studies have shown a significant association between screen time and MetS [29], [30]. In the US National Health and Nutrition Examination Survey 1999–2004 [30], the screen time was associated with increased likelihood of MetS in a dose-dependent manner independent of physical activity. The significant relation between excessive screen time and MetS in our study is troubling given that a high proportion of participants spent at least two hours per day spent on screen. In our study screen time is significantly associated with MetS, where as physical activity is not. The relationship between sedentary behavior and the metabolic syndrome may be independent of physical activity as demonstrated by a number of other studies [31].

While the definition of MetS has been criticized, it is undeniable that cardiovascular disease risk factors tend to cluster in people and that the MetS appears to be a useful aggregate measure of risk for comparisons between populations. Since it was a cross sectional study, we can only speculate about the causal pathways linking the risk factors and metabolic syndrome. Measures of subcutaneous adiposity were not available as the skin fold thickness was not measured in this study. The use of self-reported questionnaires might lead to reporting and recall bias which could influence the results. We had also limited dietary questionnaire to understand participant’s dietary habits. In additions, the data presented here might not be a good be a good reflection of the country. Nonetheless, the prevalence of obesity noted in this study is similar to the data obtained from a nation-wide study in UAE [6].

In summary, our study found that the prevalence of Met S according to the IDF criteria was 13%. The most prevalent component of MetS was low HDL cholesterol, followed by an increased waist circumference. The major significant correlates of MetS were being male, overweight, and obese and spending more than two hours on screen in a day. The higher prevalence of MetS in boys demands immediate intervention, given the potential for these children to develop chronic disease.

## Supporting Information

Table S1 **The body mass index (kg/m^2^) cut-off points used for classifying adolescents as overweight or obese from Cole^11^.**
(DOCX)
