# Supplementary material for: Metabolic Syndrome among Emirati Adolescents: A School-Based Study
Source: PLoS One. 2013 Feb 13;8(2):e56159. doi: 10.1371/journal.pone.0056159 (PMC3572014; doi:10.1371/journal.pone.0056159)
Supplement: Table S1 — The body mass index (kg/m2) cut-off points used for classifying adolescents as overweight or obese from Cole11. (DOCX) [file pone.0056159.s001.docx]

|  |  | **Overweight cut-off BMI greater than or equal to:** | |  | **Obese cut-off BMI greater than or equal to:** | |
| --- | --- | --- | --- | --- | --- | --- |
|  |  |  |  |  |  |  |
| **Age (years)** |  | **Boys** | **Girls** |  | **Boys** | **Girls** |
| 12 |  | 21.22 | 21.68 |  | 26.02 | 26.67 |
| 12.5 |  | 21.56 | 22.14 |  | 26.43 | 27.24 |
| 13 |  | 21.91 | 22.58 |  | 26.84 | 27.76 |
| 13.5 |  | 22.27 | 22.98 |  | 27.25 | 28.2 |
| 14 |  | 22.62 | 23.34 |  | 27.63 | 28.57 |
| 14.5 |  | 22.96 | 23.66 |  | 27.98 | 28.87 |
| 15 |  | 23.29 | 23.94 |  | 28.3 | 29.11 |
| 15.5 |  | 23.6 | 24.17 |  | 28.6 | 29.29 |
| 16 |  | 23.9 | 24.37 |  | 28.88 | 29.43 |
| 16.5 |  | 24.19 | 24.54 |  | 29.14 | 29.56 |
| 17 |  | 24.46 | 24.7 |  | 29.41 | 29.69 |
| 17.5 |  | 24.73 | 24.85 |  | 29.7 | 29.84 |
| 18+ |  | 25 | 25 |  | 30 | 30 |
